# Kinematic Validation of a Multi-Kinect v2 Instrumented 10-Meter Walkway for Quantitative Gait Assessments

**DOI:** 10.1371/journal.pone.0139913

**Published:** 2015-10-13

**Authors:** Daphne J. Geerse, Bert H. Coolen, Melvyn Roerdink

**Affiliations:** 1 MOVE Research Institute Amsterdam, Department of Human Movement Sciences, VU University Amsterdam, Amsterdam, The Netherlands; 2 Department of Neurology, Leiden University Medical Center, Leiden, The Netherlands; University of Toronto, CANADA

## Abstract

Walking ability is frequently assessed with the 10-meter walking test (10MWT), which may be instrumented with multiple Kinect v2 sensors to complement the typical stopwatch-based time to walk 10 meters with quantitative gait information derived from Kinect’s 3D body point’s time series. The current study aimed to evaluate a multi-Kinect v2 set-up for quantitative gait assessments during the 10MWT against a gold-standard motion-registration system by determining between-systems agreement for body point’s time series, spatiotemporal gait parameters and the time to walk 10 meters. To this end, the 10MWT was conducted at comfortable and maximum walking speed, while 3D full-body kinematics was concurrently recorded with the multi-Kinect v2 set-up and the Optotrak motion-registration system (i.e., the gold standard). Between-systems agreement for body point’s time series was assessed with the intraclass correlation coefficient (ICC). Between-systems agreement was similarly determined for the gait parameters’ walking speed, cadence, step length, stride length, step width, step time, stride time (all obtained for the intermediate 6 meters) and the time to walk 10 meters, complemented by Bland-Altman’s bias and limits of agreement. Body point’s time series agreed well between the motion-registration systems, particularly so for body points in motion. For both comfortable and maximum walking speeds, the between-systems agreement for the time to walk 10 meters and all gait parameters except step width was high (ICC ≥ 0.888), with negligible biases and narrow limits of agreement. Hence, body point’s time series and gait parameters obtained with a multi-Kinect v2 set-up match well with those derived with a gold standard in 3D measurement accuracy. Future studies are recommended to test the clinical utility of the multi-Kinect v2 set-up to automate 10MWT assessments, thereby complementing the time to walk 10 meters with reliable spatiotemporal gait parameters obtained objectively in a quick, unobtrusive and patient-friendly manner.

## Introduction

Walking speed is associated with falls [1–3], adverse events [4,5] and life expectancy [6] in older adults. A standardized clinical test often used to assess walking speed is the 10-meter walking test (10MWT). However, the 10MWT only provides a single performance measure (i.e., walking speed derived from the time to walk 10 meters), reflecting just one aspect of walking ability. To yield a more comprehensive evaluation of walking ability, quantitative gait assessments (e.g., step length, cadence and step width) may be conducted using high-end motion-registration systems. Yet, even the best motion-registration systems yield limitations when conducting quantitative gait assessments in clinical settings (e.g., costs, patient-preparation time, calibration procedures, marker occlusion, and delays in availability of results [7]).

**Fig 1:**
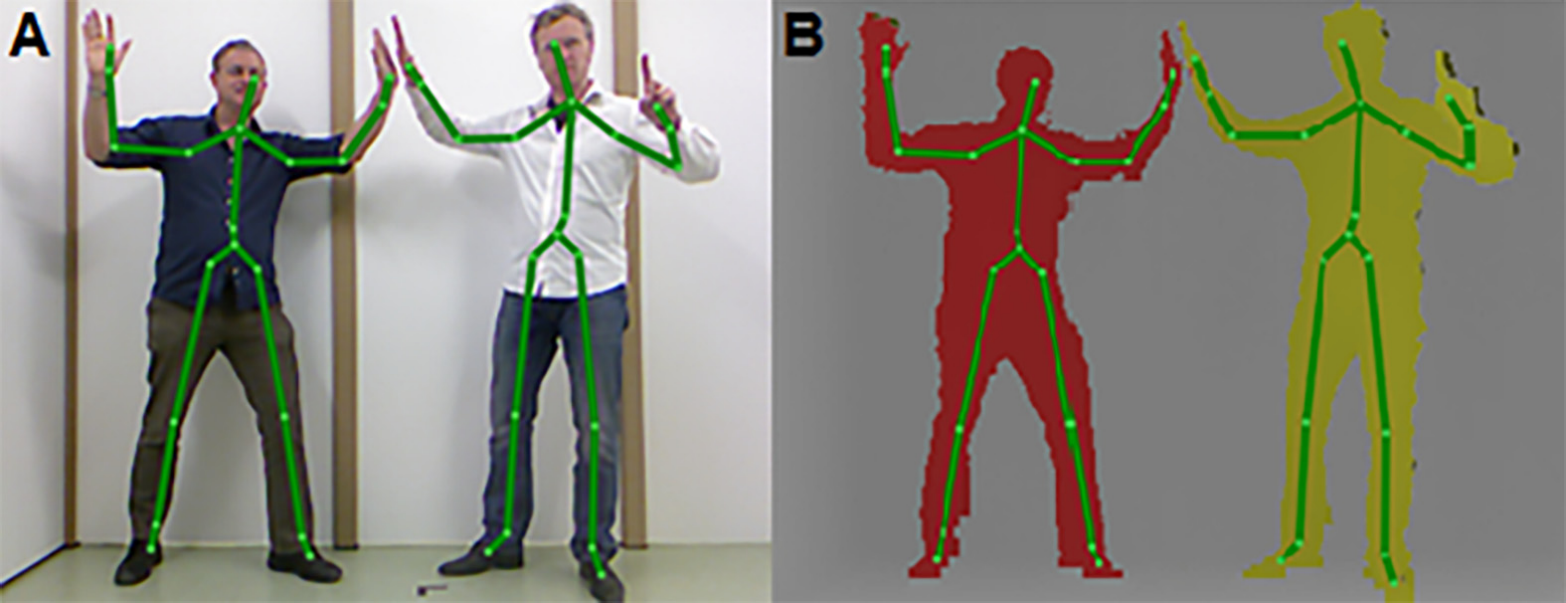
Body points derived with the human-pose estimation software of Kinect v1. (A) RGB image and (B) depth image with the corresponding body points derived with the human-pose estimation software of Kinect v1.

A promising motion-registration system to instrument the 10MWT is the Microsoft Kinect sensor, a RGB-D camera that was launched in 2011 in combination with a Software Development Kit for 3D human-pose estimation, originating from the gaming industry [8]. The development of 3D human-pose estimation software, using a large and highly varied training dataset of paired depth images and ground truth body parts to train very deep decision forests for efficient and accurate body part recognition [8], was a major undertaking by Microsoft. It successfully eliminated the need for markers and calibration procedures, thereby enabling fast and patient-friendly 3D full-body motion registration (Fig 1). This motion-registration system has gained enormous interest from developers and scientists in the context of assessment and rehabilitation of balance, posture and gait (e.g., [9–18]), since it allows for motion registration in a quick and affordable manner. Recently, the second generation of the Kinect sensor has been introduced. Key differences with the previous Kinect v1 sensor are that the Kinect v2 sensor is a time-of-flight camera with an increased resolution of the depth image, a wider field of view and improved body point tracking [19], possibly leading to improved results.

Several studies have demonstrated that spatiotemporal gait parameters can be validly obtained using a single Kinect v1 sensor [9,11,13,14,17], and recently also for a single Kinect v2 sensor [15]. However, these studies only analyzed a few steps since accurate body point tracking with the Kinect sensor is only possible between 0.8 and 4.0 meters from the Kinect v1 sensor and between 0.5 and 4.5 meters from the Kinect v2 sensor due to the limited field of view and poorer depth-image quality at greater distances. One way to cover a larger volume, such as the walkway of the 10MWT, is to use multiple spatially and temporally integrated Kinect sensors. Hereby measurement volume may be increased, while preserving good quality depth images for accurate body point tracking. This supposedly allows for the parametrization of a large number of steps during walking from high quality 3D body point’s time series. In view of Kinect’s v2 higher resolution depth images, improved body point tracking and enlarged area for accurate body point tracking, the current study will explore the potential of a multi-Kinect v2 set-up for instrumenting the 10MWT.

The objective of this study is to determine the usability of a multi-Kinect v2 set-up to quantitatively assess gait during the 10MWT. Because the multi-Kinect v2 set-up has not yet been validated for 3D full-body motion registration, its performance will be compared to a gold standard in 3D measurement accuracy (i.e., the Optotrak active-marker 3D optical tracking system, Northern Digital Inc., Waterloo, Canada). The between-systems agreement will be examined for raw data (i.e., body point’s time series) and spatiotemporal gait parameters (e.g., step length, cadence and step width). In addition, the between-systems agreement for the performance measure of the 10MWT (i.e., time to walk 10 meters) will be assessed between the multi-Kinect v2 set-up, the Optotrak motion-registration system (i.e., the gold-standard reference) and the stopwatch (i.e., the clinical standard).

## Methods

### Subjects

A heterogeneous group of 21 healthy subjects in terms of gender (11 males, 10 females), age (mean [range]: 30.2 [19–63] years), height (176.1 [158–190] cm) and weight (70.5 [53–83] kg) took part in this experiment. Subjects did not have any medical condition that would influence walking.

### Ethics statement

The current study was approved by the ethics committee of the Department of Human Movement Sciences (VU University Amsterdam, Amsterdam). All subjects provided written informed consent prior to participation. The subjects in Fig 1 have given written informed consent, as outlined in the PLOS consent form, to publish this photograph.

### Experimental set-up and procedure

**Fig 2:**
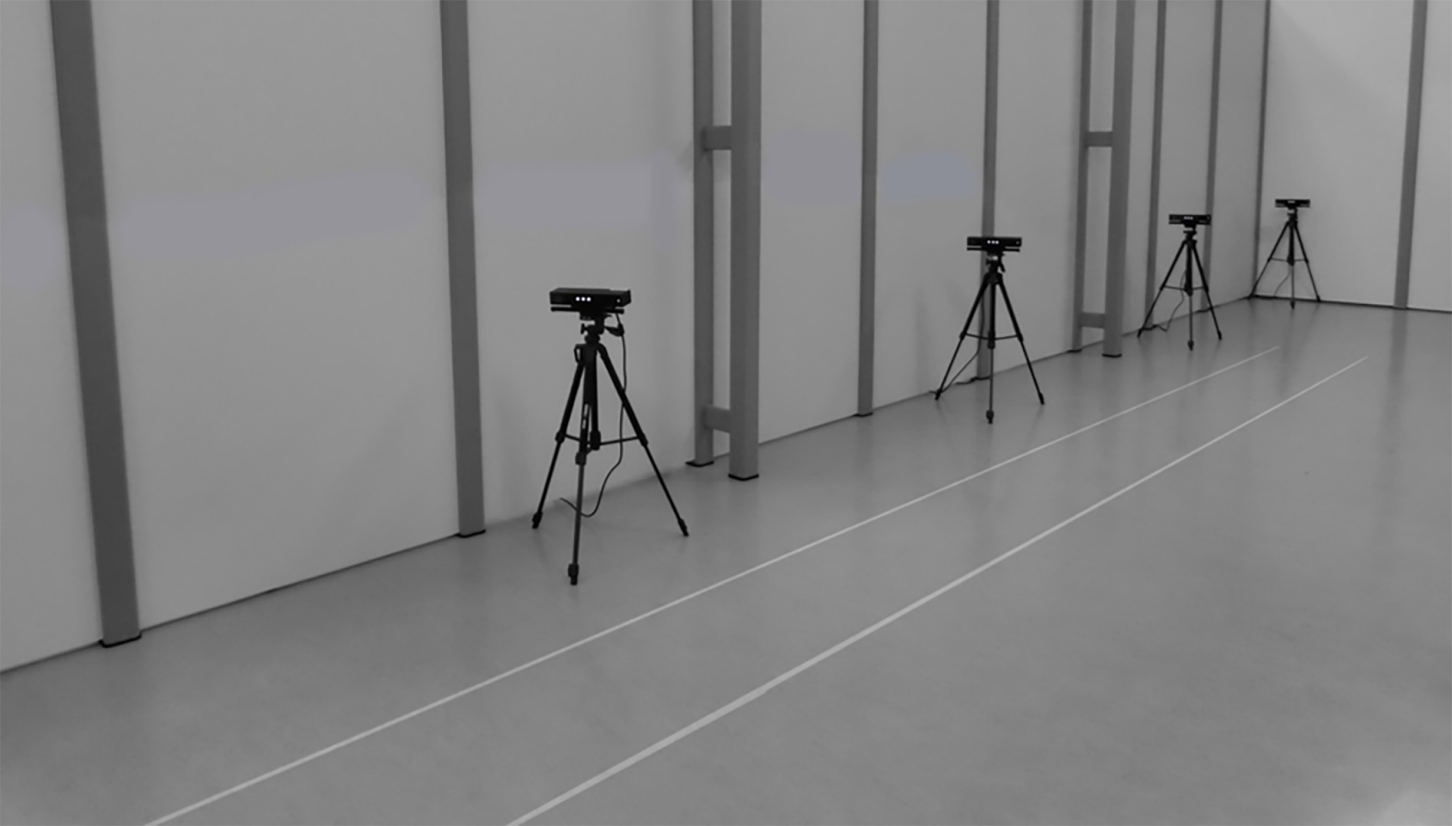
Overview of the multi-Kinect v2 set-up.

Full-body kinematics was recorded with four spatially and temporally integrated Microsoft Kinect v2 sensors and the Optotrak system (Northern Digital Inc., Waterloo, Canada). The multi-Kinect v2 set-up is displayed in Fig 2. The four Kinect v2 sensors were positioned on tripods alongside a walkway of 10 by 0.5 meters at a height of 0.75 meters. The sensors were placed 0.5 meters from the left border of the walkway with an angle of 70 degrees relative to the walkway direction. The first sensor was positioned at 4 meters from the start of the walkway. The other three sensors were placed at inter-sensor distances of 2.5 meters. In addition, five Optotrak cameras (i.e., a combination of two Optotrak 3020 and three Optotrak Certus cameras, which are all compatible with each other) were positioned around the walkway to cover the same area as the multi-Kinect v2 set-up. The so-obtained Optotrak set-up ensured sub-millimeter accuracy throughout the 10-meter walkway. The coordinate systems of the multi-Kinect v2 set-up and the Optotrak system were aligned using a spatial calibration grid.

**Fig 3:**
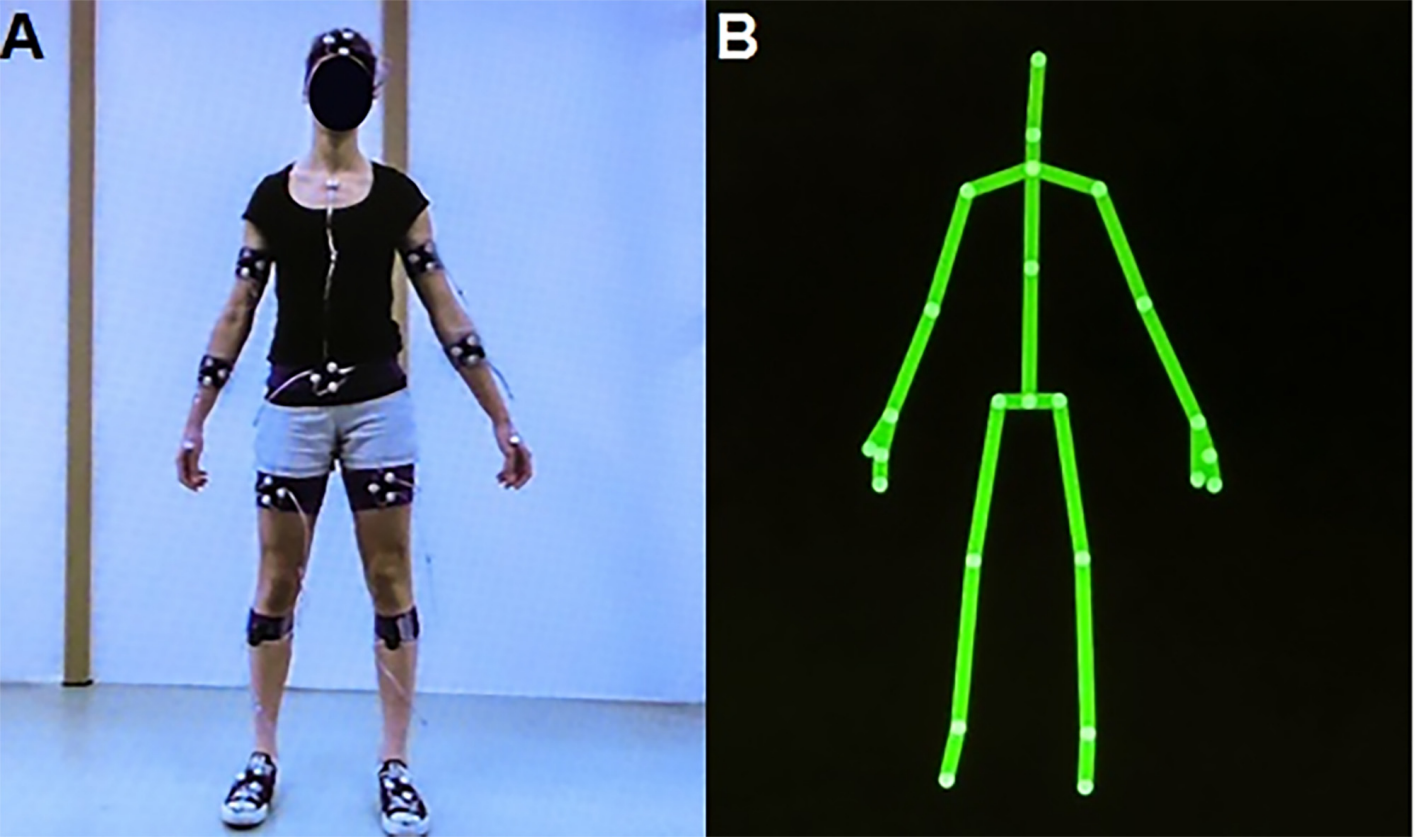
Body point determination with the Optotrak and Kinect v2 systems. (A) Subject with all markers of the Optotrak system; (B) Same subject with body points derived with the human-pose estimation algorithm of Kinect v2.

The Kinect for Windows Software Development Kit (SDK 2.0, www.microsoft.com) provides, with a sampling rate of 30 Hz, the 3D positions of 25 body points (Fig 3B). These body points are: head, neck, spine shoulder, spine mid, spine base and right and left shoulder, elbow, wrist, hand, thumb, hand tip, hip, knee, ankle and foot. For motion registration with the Optotrak system (Northern Digital Inc., Waterloo, Canada, using First Principles data acquisition software with a sampling rate of 60 Hz), subjects were asked to wear tight-fitting shorts and a t-shirt to limit clothing-related marker occlusion. Smart Marker Rigid Bodies (Northern Digital Inc., Waterloo, Canada) were attached to the head, upper arms, forearms, lower abdomen, upper legs, lower legs and feet (Fig 3A), allowing for 6 degrees of freedom tracking of body segments. In addition, 30 anatomical landmarks were digitized using a 3-marker digitizing probe to define various body point positions (so-called virtual markers) on abovementioned body segments. Smart markers were also placed on the sternum, hands and feet. The body points represented by Optotrak’s virtual markers and/or smart markers were selected to closely match Kinect’s body points (see S1 Table), although sometimes arbitrary positional differences between the body point’s time series of the two motion-registration systems could not be prevented because 1) the exact definitions of the body points given by the human-pose estimation algorithms of Kinect v2 are not known and 2) virtual markers and smart markers are by definition positioned at the contours of the body while Kinect v2 body points are typically estimated within the body. For example, the smart marker representing Kinect’s spine shoulder was placed on the sternum (see S1 Table), which deviates in AP direction from the within-body spine shoulder given by the human-pose estimation algorithm of Kinect v2, thus resulting in a between-systems positional mismatch. Positions of the neck, spine mid, thumbs and hand tips body points were not tracked with the Optotrak system due to the limited number of available smart markers, rendering a total of 19 out of aforementioned 25 body points eligible for a between-systems agreement analysis (as specified in S1 Table).

Before conducting the experiment, the quality of depth image of the subject was checked since some textiles are known to corrupt the infrared radiation emitted by the previous Kinect v1 sensor, making human-pose estimation less accurate [17]. No problems were encountered with clothing of the subjects, possibly owing to the improved properties of the Kinect v2 sensor. Subsequently, subjects performed the 10MWT at two different walking speeds, namely comfortable walking speed (CWS) and maximum walking speed (MWS). Both conditions were performed three times in a fixed order (i.e., three times CWS followed by three times MWS). Subjects were instructed to start walking at the fourth, high-pitched beep of a standardized auditory start command (i.e., three low-pitched beeps followed by one high-pitched beep) and to continue walking until they had fully crossed the finish line. The standardized auditory start command was synchronized with the multi-Kinect v2 set-up. Synchronization between the two motion-registration systems was achieved by a synchronization movement (i.e., ab- and adduction of both arms) that participants performed prior the auditory start command of each trial. Motion registration started before the synchronization movement and ended well after the subject had passed the 10-meter line. Time to walk 10 meters (i.e., from final beep onset until the moment that the most forward ankle passed the 10-meter line, according to the recommendations of Graham et al. [20]) was determined using a stopwatch. A video showing body point’s time series simultaneously for both measurement systems during the 10MWT is available in S1 Video. This video also includes the synchronization movement and the standardized auditory start command.

### Data pre-processing

The 3D positional data of body points were first pre-processed per Kinect sensor separately. Inferred body points (i.e., when a body point was not visible due to for example occlusion, Kinect’s human-pose estimation software inferred its position) were considered as missing values. Moreover, since the sampling frequency of the Kinect system is not constant (i.e., apart from 20 outliers in inter-sample intervals for multiple subjects but confined to one Kinect sensor, the remaining inter-sample intervals ranged from 32 to 34 ms), the body point’s time series were linearly interpolated using Kinect’s timestamps to ensure a constant sampling frequency of 30 Hz, without filling in the parts with missing values. Data points not adhering to the requirements for valid human-pose estimation (e.g., minimum of 15 tracked body points out of the 25 body points, tracked data points for the head and at least one foot and no outliers in segment lengths) were removed from the time series. Subsequently, data of the four Kinect sensors were combined by taking for each sample the 3D positions of the body points of a validly estimated human pose. If, for a given sample, more than one sensor contained valid human-pose data, the associated body point’s 3D positions were averaged for that specific sample. Optotrak data were down-sampled to 30 Hz. Subsequently, the cross-covariance and time lag were determined for paired time series in the mediolateral (ML) and vertical (V) direction of the elbows, wrists and hands during the synchronization movement. These time series were first interpolated with a spline algorithm in case of missing data. The median of the time lags was used to temporally align the time series of the two motion-registration systems. Time-synchronized 3D body point’s time series of both systems are presented as supplementary material, starting from final beep onset until the moment that for both systems the most forward ankle passed the 10-meter line (see S1 Data). Body point’s time series with more than 50 percent of missing values were excluded from further analyses. No time series were excluded for the multi-Kinect v2 set-up, whereas 17 out of 2394 time series were excluded for Optotrak, including two time series of the ankles from which gait parameters were derived. The missing values of the remaining data were interpolated with a spline algorithm. The so-obtained time series were used for assessing the between-systems agreement in body point’s time series (see Data analysis) and for the quantification of several gait parameters, as specified in the next paragraph.

**Fig 4:**
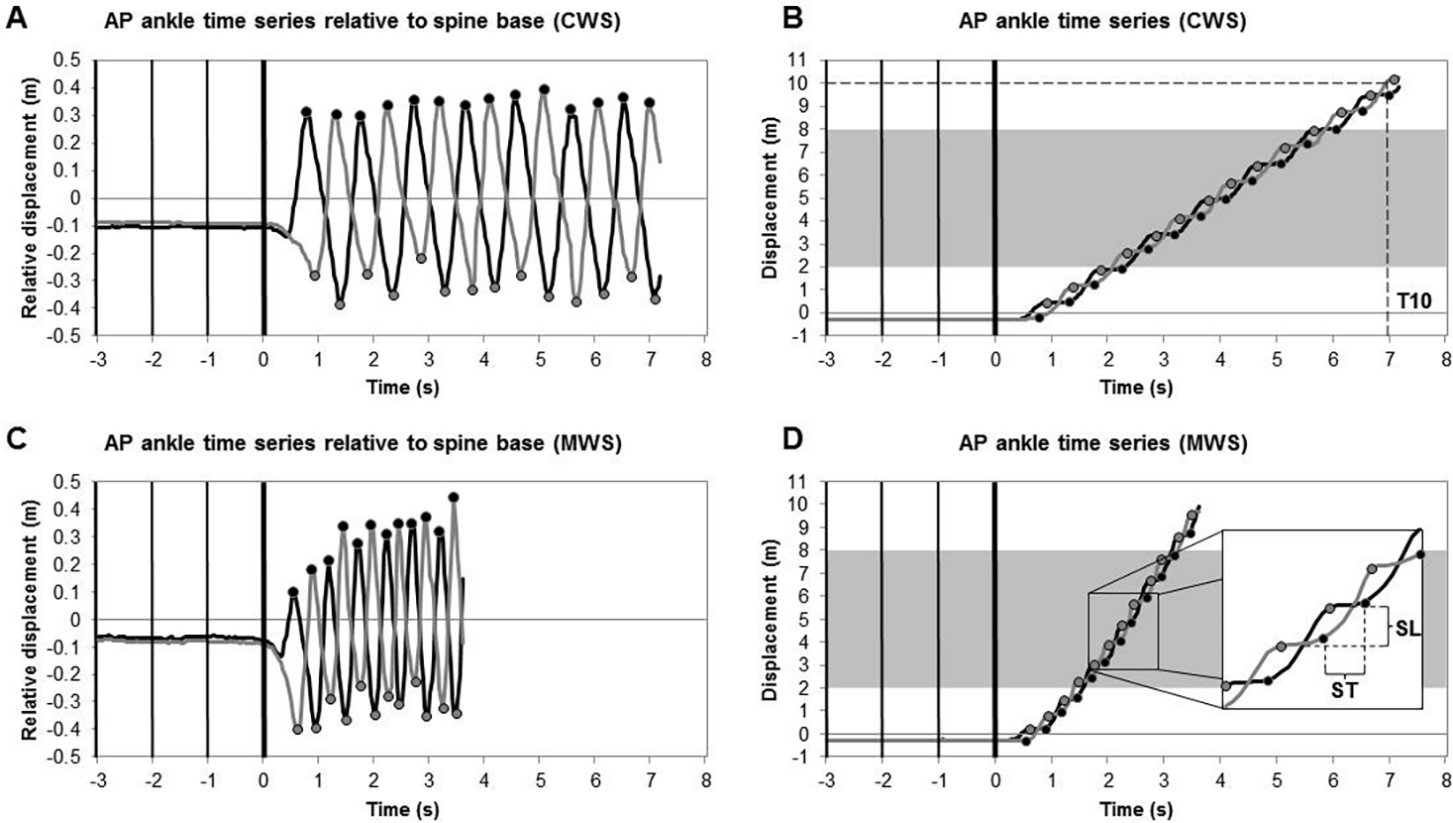
Overview of the analysis of spatiotemporal gait parameters. Analyses for CWS (panels A and B) and MWS (panels C and D) conditions are based on AP displacement data of the right (gray lines) and left (black lines) ankles as a function of time for the multi-Kinect v2 set-up. AP ankle time series relative to the spine base (panels A and C) were used to estimate instants of foot contact (black dots) and foot off (gray dots) for each step. Step location was defined as the median value of the AP ankle time series during the single-support stance phase (i.e., the horizontal plateaus delimited by foot off and foot contact events of the contralateral foot in panels B and D). Vertical bars represent the four beep onsets of the auditory start command. The shaded area in panels B and D represent the 6-meter window from which spatiotemporal gait parameters were derived. Dashed lines in panels B and D schematically define the time to walk 10 meters (T10), step time (ST) and step length (SL).

Several gait parameters were calculated from the body point’s time series, separately for both measurement systems. The following spatiotemporal gait parameters were all determined for the intermediate 6 meters (i.e., from the 2-meter to the 8-meter line), reducing the effect of gait acceleration and deceleration on the gait parameters [21]. Walking speed (in cm/s) was defined as the distance travelled between the 2-meter and 8-meter line on the walkway divided by the time, using the data of the spine shoulder. For the other gait parameters, estimates of foot contact and foot off were required, stemming from respectively the maxima and minima of the anterior-posterior (AP) time series of the ankles relative to that of the spine base [22] (Fig 4A and 4C). For spatial gait parameters, first right and left step locations were determined, defined as the median value of the right and left ankle position in the AP and ML direction during the respective single-support stance phases (i.e., between foot off and foot contact of the contralateral foot). Based on these AP and ML step locations, various spatial gait parameters were determined. Step length (in cm) was calculated as the AP difference of consecutive step locations (Fig 4D). Stride length (in cm) was calculated as the AP difference of consecutive ipsilateral step locations. Moreover, step width (in cm) was estimated by taking the absolute ML difference of consecutive step locations. Cadence (in steps/min) was calculated from the number of steps in the time interval between the first and last estimate of foot contact. Step time (in s) was calculated as the time interval between two consecutive instants of foot contact (Fig 4D). Consequently, stride time (in s) was calculated as the time interval between two consecutive ipsilateral instants of foot contact. For step length, stride length, step width, step time and stride time, median values within the 6-meter window were used as outcome measures per trial since Baldewijns et al. [9] demonstrated superior agreement between registration systems on a per walk basis.

The performance measure of the 10MWT, that is the time to walk 10 meters (in s), was defined as the time from final beep onset until the moment that the most forward ankle passed the 10-meter line (Fig 4B). For comparison with the stopwatch score, serving as the clinical reference, the time to walk 10 meters was also determined from data of the multi-Kinect v2 set-up and the Optotrak system, the latter serving as the gold-standard reference.

### Data analysis

First, the between-systems agreement was calculated for the body point’s time series from final beep onset until the moment that the most forward ankle passed the 10-meter line. For the AP direction, the trend was removed using a bidirectional, second-order Butterworth high-pass filter (cutoff frequency of 0.5 Hz) to reduce the effect of a large within-subject variation (increasing from 0 to 10 meter) on the agreement statistic, which would become arbitrarily high [23]. The agreement between the time series of the two motion-registration systems was calculated for each body point in the AP, ML and V direction by means of the intraclass correlation coefficient for consistency (ICC_(C,1)_; [24]). We selected ICC_(C,1)_ in view of abovementioned somewhat arbitrary between-systems mismatches in body point’s time series (see S1 Table). The average ICC_(C,1)_ was constructed over all trials per system, body point and direction for each subject. From these values, the average ICC_(C,1)_ over subjects was calculated for each system, body point and direction, including confidence intervals.

Second, the between-systems agreement for spatiotemporal gait parameters was calculated. Spatiotemporal gait parameters were based on specific within-system time series’ features (e.g., minima or maxima, consecutive step locations) and hence less susceptible to arbitrary systematic between-systems positional differences in body point’s time series. Therefore, the ICC for absolute agreement (ICC_(A,1)_; [24]) was selected. The agreement in the time to walk 10 meters obtained with the multi-Kinect v2 set-up, the Optotrak system (gold standard) and a stopwatch (clinical standard) was also assessed using ICC_(A,1)_.

In line with Cicchetti [25], we regard ICC values above 0.60 as good and ICC values above 0.75 as excellent. ICC_(A,1)_ values were complemented by mean differences and precision values obtained with a Bland-Altman analysis (i.e., the bias and the limits of agreement, respectively; [26]). Since large differences were expected between CWS and MWS conditions for all gait parameters, leading to large within-subject variation that would arbitrarily inflate the between-systems agreement [23], the agreement for gait parameters and time to walk 10 meters was analyzed separately for both conditions. In line with Flansbjer et al. [27], the average time to walk 10 meters was constructed over the three trials per condition per subject. For the spatiotemporal gait parameters the average was hence also constructed over the three trials per condition per subject. For each condition, at least two trials had to be valid (i.e., less than 50 percent of missing values and, for the time to walk 10 meters, data around the 10-meter line and no error in pressing the stopwatch) in order to compute the average over the trials. This resulted in the exclusion of one subject for further analysis of the between-systems agreement for the time to walk 10 meters for the MWS condition.

## Results

### Agreement between body point’s time series

**Fig 5:**
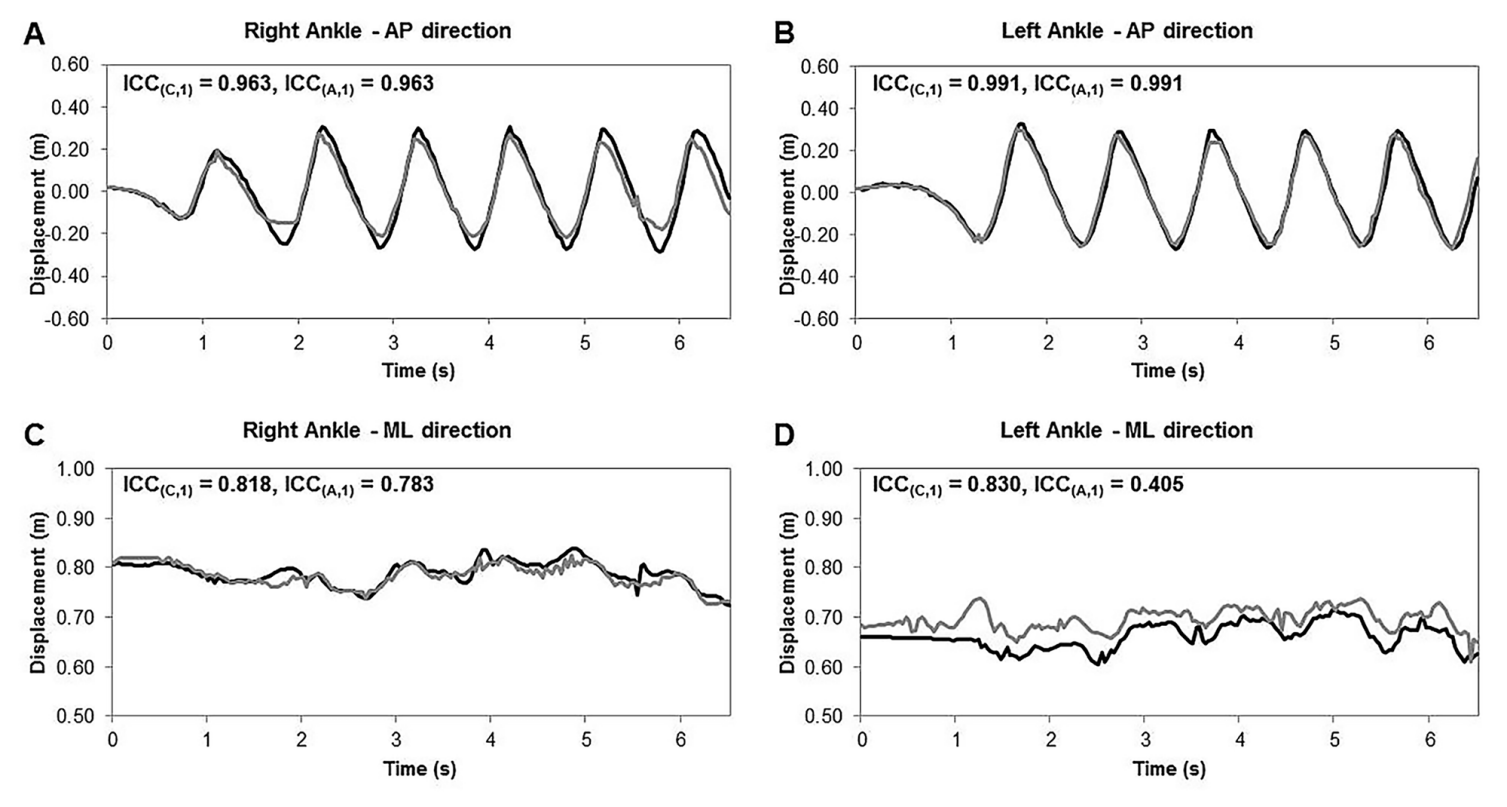
Representative ankle time series of the multi-Kinect v2 set-up and the Optotrak system. Multi-Kinect v2 (gray lines) and Optotrak (black lines) time series of the right (panels A and C) and left (panels B and D) ankle in the AP (detrended) and ML direction for a part of a CWS trial, including between-systems agreement assessed with the intraclass correlation coefficient for consistency (ICC_(C,1)_) and absolute agreement (ICC_(A,1)_).

The agreement (ICC_(C,1)_) between the body point’s time series of the multi-Kinect v2 set-up and the gold-standard Optotrak motion-registration system for all 19 matched body points in AP (detrended), ML and V directions are listed in Table 1. Apart from the hips, there was a good to excellent agreement in body point’s time series between the two motion-registration systems in the AP direction. Furthermore, all gait parameters were derived from time series with high (i.e., ML time series of the right ankle) or excellent levels of agreement (all other time series), as highlighted in Table 1 (bold values). Fig 5 shows an example of a part of the AP (detrended) and ML time series of the right and left ankle for the multi-Kinect v2 set-up and the Optotrak system during a CWS trial with corresponding ICC_(C,1)_ values (as well as ICC_(A,1)_ values to illustrate the effect of a systematic between-systems mismatch in body point’s time series on ICC values).

**Table 1:** Agreement statistics for body point’s time series.

	AP	ML	V
**Head**	0.736 (0.709–0.762)	0.753 (0.714–0.792)	0.832 (0.801–0.863)
**Spine shoulder**	**0.777 (0.747–0.808)**	0.744 (0.709–0.780)	0.870 (0.850–0.890)
**Spine base**	**0.864 (0.852–0.877)**	0.824 (0.797–0.850)	0.790 (0.752–0.828)
**Left shoulder**	0.746 (0.671–0.821)	0.734 (0.658–0.810)	0.824 (0.740–0.908)
**Left elbow**	0.917 (0.847–0.987)	0.764 (0.685–0.842)	0.567 (0.488–0.646)
**Left wrist**	0.970 (0.961–0.980)	0.903 (0.884–0.922)	0.879 (0.853–0.906)
**Left hand**	0.973 (0.966–0.980)	0.903 (0.882–0.923)	0.900 (0.880–0.921)
**Right shoulder**	0.787 (0.761–0.813)	0.751 (0.712–0.790)	0.849 (0.813–0.885)
**Right elbow**	0.936 (0.919–0.953)	0.794 (0.760–0.828)	0.628 (0.569–0.688)
**Right wrist**	0.939 (0.908–0.971)	0.850 (0.787–0.914)	0.773 (0.711–0.834)
**Right hand**	0.911 (0.868–0.953)	0.828 (0.763–0.893)	0.693 (0.622–0.763)
**Left hip**	0.479 (0.418–0.540)	0.736 (0.693–0.779)	0.572 (0.506–0.637)
**Left knee**	0.942 (0.922–0.963)	0.786 (0.739–0.833)	0.221 (0.152–0.289)
**Left ankle**	**0.970 (0.955–0.984)**	**0.871 (0.844–0.898)**	0.392 (0.342–0.442)
**Left foot**	0.923 (0.866–0.980)	0.842 (0.781–0.904)	0.443 (0.396–0.491)
**Right hip**	0.386 (0.308–0.465)	0.749 (0.709–0.789)	0.616 (0.571–0.661)
**Right knee**	0.847 (0.804–0.890)	0.587 (0.525–0.650)	0.163 (0.128–0.198)
**Right ankle**	**0.911 (0.891–0.932)**	**0.744 (0.708–0.781)**	0.198 (0.133–0.262)
**Right foot**	0.819 (0.786–0.852)	0.685 (0.641–0.729)	0.279 (0.234–0.325)

Between-systems agreement (ICC_(C,1)_ with 95% CI) for body point’s time series in AP (detrended), ML and V directions. Bold values represent agreement for time series from which spatiotemporal gait parameters were derived.

Abbreviations: ICC_(C,1)_ = intraclass correlation coefficient for consistency; CI = confidence interval; AP = anterior-posterior; ML = mediolateral; V = vertical.

### Agreement of spatiotemporal gait parameters

The agreement statistics of the spatiotemporal gait parameters are presented in Table 2. Apart from step width, the between-systems agreement for spatiotemporal gait parameters was excellent for CWS (ICC_(A,1)_ ≥ 0.888) and MWS (ICC_(A,1)_ ≥ 0.951) conditions. This was supported by relatively small biases and narrow limits of agreement (Table 2). Step width showed a good between-systems agreement (CWS: 0.646, MWS: 0.705) with proportionally higher biases and wider limits of agreement (Table 2). Bland-Altman plots for spatiotemporal gait parameters are available in S1 Fig.

**Table 2:** Agreement statistics for spatiotemporal gait parameters.

		Multi-Kinect v2 set-up	Optotrak system		
		Mean ± SD	Mean ± SD	Bias (95% LoA)	ICC_(A,1)_
**Walking speed (cm/s)**	**CWS**	142.8 ± 11.7	143.9 ± 11.8	1.1 (0.1 2.1)	0.995
	**MWS**	220.2 ± 32.2	220.8 ± 31.7	0.6 (-1.4 2.6)	0.999
**Cadence (steps/min)**	**CWS**	115.9 ± 6.2	115.0 ± 5.9	-0.9 (-3.0 1.2)	0.974
	**MWS**	147.8 ± 21.9	145.7 ± 21.7	-2.1 (-7.4 3.3)	0.988
**Step length (cm)**	**CWS**	75.5 ± 5.7	75.4 ± 5.7	-0.1 (-1.4 1.2)	0.994
	**MWS**	92.5 ± 8.0	92.5 ± 7.8	-0.1 (-2.1 2.0)	0.992
**Stride length (cm)**	**CWS**	151.0 ± 11.3	151.1 ± 11.2	0.1 (-0.7 0.9)	0.999
	**MWS**	185.6 ± 15.7	185.4 ± 15.6	-0.1 (-1.6 1.4)	0.999
**Step width (cm)**	**CWS**	11.3 ± 2.1	10.0 ± 3.1	-1.3 (-5.2 2.6)	0.646
	**MWS**	12.1 ± 2.4	10.6 ± 3.4	-1.5 (-5.2 2.2)	0.705
**Step time (s)**	**CWS**	0.52 ± 0.03	0.52 ± 0.03	0.01 (-0.02 0.03)	0.888
	**MWS**	0.42 ± 0.05	0.42 ± 0.05	0.00 (-0.03 0.03)	0.951
**Stride time (s)**	**CWS**	1.04 ± 0.06	1.05 ± 0.06	0.01 (-0.02 0.04)	0.962
	**MWS**	0.82 ± 0.09	0.84 ± 0.10	0.01 (-0.02 0.04)	0.979

Mean values, between-subjects standard deviations (SD) and agreement statistics (bias, limits of agreement [95% LoA] and intraclass correlation coefficient for absolute agreement [ICC_(A,1)_]) for spatiotemporal gait parameters of CWS and MWS conditions.

Abbreviations: CWS = comfortable walking speed; MWS = maximum walking speed.

### Agreement of time to walk 10 meters

**Fig 6:**
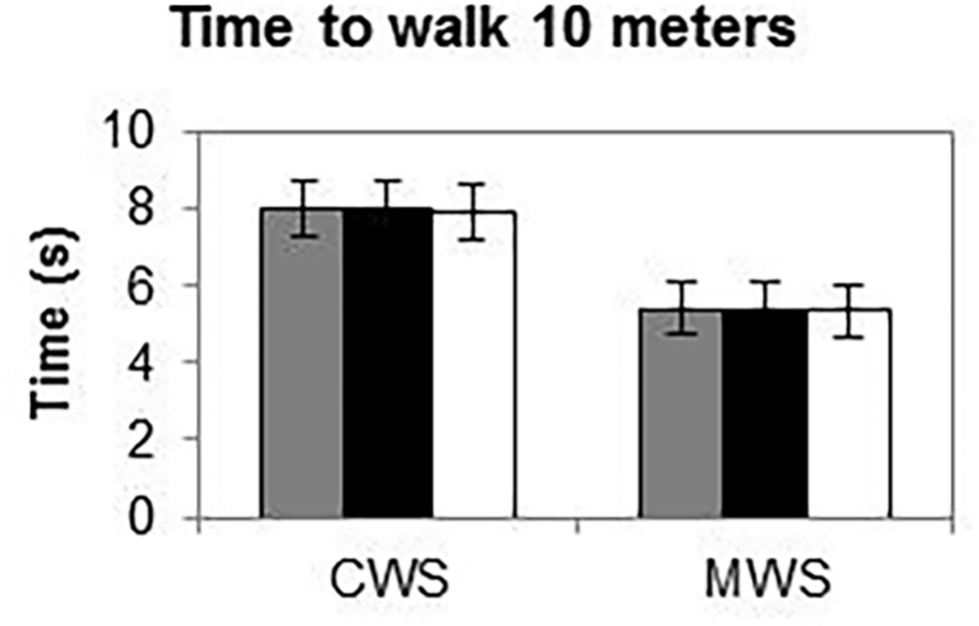
Time to walk 10 meters for CWS and MWS conditions. Bars represent average time to walk 10 meters for the multi-Kinect v2 set-up (gray bars), the Optotrak motion-registration system as the gold-standard reference (black bars) and the stopwatch as the clinical standard (white bars).

Mean values of the time to walk 10 meters for CWS and MWS conditions are presented in Fig 6. There was a high level of agreement between the measurement systems according to the ICC_(A,1)_ for both conditions. For the multi-Kinect v2 set-up and the Optotrak system, ICC_(A,1)_ values were excellent for CWS (ICC_(A,1)_ = 0.998) and MWS (ICC_(A,1)_ = 0.999), with biases being smaller than one sample (CWS: -0.01 s, MWS: -0.01 s) and narrow limits of agreement (CWS: [-0.11 0.09] s, MWS: [-0.07 0.06] s). The comparison between the multi-Kinect v2 set-up and the stopwatch also revealed excellent ICC_(A,1)_ values (CWS: 0.988, MWS: 0.989), but biases were greater (CWS: -0.09 s, MWS: -0.08 s) and limits of agreement wider (CWS: [-0.23 0.05] s, MWS: [-0.21 0.06] s). The same was true for the comparison between the Optotrak system and the stopwatch: excellent ICC_(A,1)_ values (CWS: 0.987, MWS: 0.990) but biases were approximately two samples (CWS: -0.08 s, MWS: -0.07 s) and limits of agreement were again wider (CWS: [-0.26 0.11] s, MWS: [-0.21 0.07] s).

## Discussion

In the current study, we evaluated a multi-Kinect v2 set-up for quantitative gait assessment during the 10MWT by determining between-systems agreement for body point’s time series, for spatiotemporal gait parameters and for the time to walk 10 meters. Performance of the multi-Kinect v2 set-up was compared to the Optotrak system (i.e., the gold-standard reference) to validate 3D full-body kinematical data of the just-released Kinect v2 sensor. We observed a good to excellent agreement between the two motion-registration systems for raw data (i.e., relevant body point’s time series), spatiotemporal gait parameters and the time to walk 10 meters.

To the best of our knowledge, this study is the first to statistically compare unfiltered body point’s time series stemming from a multi-Kinect v2 set-up to a gold-standard reference. Covering the entire measurement volume with a marker-based motion-registration system was quite difficult and required many cameras to avoid marker occlusion. In fact, the number of excluded body point’s time series due to excessive missing values was substantially larger for the marker-based gold standard in 3D measurement accuracy (17 excluded time series, average percentage of missing values was 6.8%) than for the multiple-Kinect v2 set-up (no excluded time series, average percentage of missing values was 5.0%). For the remaining 2377 time series, ICC_(C,1)_ values were generally exceeding 0.60 for all directions, indicating a good to excellent between-systems agreement. Nevertheless, some time series only demonstrated a poor to fair between-systems agreement, especially time series exhibiting a small range of motion. Note that the ICC is constructed using models that assume equal variance between two variables [24]. With a small range of motion (i.e., with low signal power and hence low true within-system variation), the noisier Kinect v2 data may have caused the error-variances of the two motion-registration systems to differ, with consequently a lower between-systems agreement. This is supported by results of a previous study [28], showing that larger movements of Parkinson’s disease patients were better tracked by a Kinect v1 sensor than smaller movements. Thus, as long as body points are moving (i.e., high signal power), the resultant time series of Kinect v2 match well with those stemming from a gold standard in 3D measurement accuracy. Furthermore, low-pass filtering time series may also increase the between-systems agreement.

In the current study, all spatiotemporal gait parameters were derived from body point’s time series with high (for the ML time series of the right ankle) or excellent levels of agreement (for all other time series; see Table 1, bold values). This resulted in excellent between-systems agreement (high ICC_(A,1)_ values) of the from these time series derived spatiotemporal gait parameters walking speed, cadence, step length, stride length, step time and stride time. These spatiotemporal gait parameters can be accurately obtained with the multi-Kinect v2 set-up, as testified by negligible biases and narrow limits of agreement (Table 2). Step width was the only gait parameter that demonstrated good instead of excellent absolute agreement (Table 2). The deviant findings for step width may be due to systematic within-subject differences in ML ankle position time series between the two motion-registration systems. An example of such a systematic positional difference is presented in Fig 5. The left ML ankle position obtained with the multi-Kinect v2 set-up was about 3 to 4 centimeters more lateral compared to Optotrak’s left ML ankle position (Fig 5D) while the right ML ankle positions matched well between the two systems (Fig 5C), resulting in a substantial bias of 3.6 cm in step width for this specific subject. This systematic between-systems mismatch for the left ML ankle position was confirmed by a clear difference between ICC values for consistency and absolute agreement (ICC_(C,1)_ = 0.830, ICC_(A,1)_ = 0.405; Fig 5D), whereas for the right ML ankle positions the ICC values were similar (ICC_(C,1)_ = 0.818, ICC_(A,1)_ = 0.783; Fig 5C). Note that this positional mismatch in ankle time series was not consistent among subjects in terms of its size, sign and side, which may explain the relatively larger between-subjects variation in the between-systems difference for step width (i.e., relatively wider limits of agreement in Table 2).

Kitsunezaki et al. [29] also assessed the possibility of instrumenting the 10MWT with multiple Kinect sensors. Specifically, they used two temporally integrated Kinect v1 sensors that were positioned at the 2-meter and 8-meter lines of a 10-meter walkway to determine the walking time of the intermediate 6 meters of the 10MWT. The mean difference in walking times obtained with the clinical standard (i.e., stopwatch) and the two Kinect v1 sensors was 0.15 seconds, which led the authors to conclude that a Kinect-based assessment was acceptable for practical use [29]. In the current study we quantified the time to walk 10 meters with a multi-Kinect v2 set-up, a gold-standard motion-registration system and a stopwatch. Despite examining walking time over a greater walking distance than Kitsunezaki et al. [29], we found smaller differences between the three measurement systems (≤ 0.09 s), especially between the multi-Kinect v2 set-up and the gold-standard motion-registration system (0.01 s). Noteworthy is that the agreement between these two motion-registration systems–in terms of ICC_(A,1)_, biases and limits of agreement–was better than the agreement of either one with the clinical standard (i.e., stopwatch). To put these findings in perspective, the between-systems differences in the time to walk 10 meters were about 30 to 300 times smaller than the within-system differences between CWS and MWS conditions. Moreover, the meaningful change in walking speed of 5 cm/s according to Perera et al. [30] is at least twice as large as the between-systems differences in walking speed observed in the current study (i.e., after transforming the time to walk 10 meters to walking speed, ≤ 2.5 cm/s).

A multi-Kinect v2 set-up, such as the one described in the current study, may in practice be employed to automate the assessment of the 10MWT. An advantage of this set-up is that the 10MWT and quantitative gait assessment can be conducted simultaneously to reduce the time needed for a comprehensive assessment of walking ability. This could be beneficial for clinical applications, especially in view of our observation that the set-up can provide reliable estimates of the time to walk 10 meters and commonly used spatiotemporal gait parameters in a very quick, unobtrusive and patient-friendly manner. Other advantages of the Kinect v2 sensor are that 3D positional data of 25 body points (of up to six persons!) are tracked and available in real time, without markers, and not requiring time-consuming pre-registration calibration and post-registration labeling/tracking. Considering these assets, one may consider a multi-Kinect v2 set-up as a serious alternative for quantitative gait assessments.

A limitation of the multi-Kinect v2 set-up is the relatively low sampling frequency of 30 Hz. Although a good agreement between the multi-Kinect v2 set-up and the Optotrak system was found for almost all outcome measures of the current study, other outcome measures of interest may require higher sampling rates (e.g., the analysis of stride-to-stride fluctuations in stride times [31]). Another limitation of the study was that the between-systems agreement was only assessed for healthy subjects. Before implementing the multi-Kinect v2 walkway in the clinic, gait parameters for the patient groups of interest should be validated first. Moreover, one can imagine that in a clinical context an accompanying person such as a therapist wants to walk along with a patient for safety reasons. Because 3D positional data of body points of up to six persons can be tracked with a Kinect v2 sensor, each being allocated with a unique body identification number, it is important to ensure the correct allocation of data to a specific person when tracking multiple persons with multiple Kinects (e.g., using minimization of 3D positional data when moving from one camera’s field of view to another). Therefore, gait parameters need to be validated in various patient groups both with and without an accompanying person. As in healthy controls, good human-pose estimation is to be expected for patients. Clark et al. [32], for example, recently concluded that gait parameters of stroke patients derived from Kinect v1 data were highly reliable and could provide valuable additional information for gait analysis alongside the 10WMT. They stated that their findings provide support for implementing Kinect-based gait assessments in clinical settings [32]. With the development and validation of the multi-Kinect v2 instrumented 10-meter walkway, the current study may help pave the way to fulfill that premise.

## Conclusion

Body point’s time series obtained with a multi-Kinect v2 set-up match well with those derived with a gold standard in 3D measurement accuracy, particularly so for body points in motion. The excellent absolute agreements with the gold standard observed for time to walk 10 meters, walking speed, cadence, step length, stride length, step time and stride time emphasize that those parameters can be reliably obtained with the multi-Kinect v2 set-up. Future studies are recommended to test the clinical utility of the multi-Kinect v2 set-up to automate 10MWT assessments, thereby complementing the time to walk 10 meters with reliable spatiotemporal gait parameters obtained objectively in a quick, unobtrusive and patient-friendly manner.

## Supporting Information

S1 Data Body point’s time series in the AP, ML and V direction for the multi-Kinect v2 set-up and the Optotrak system. (ZIP)

S1 Fig Bland-Altman plots for the spatiotemporal gait parameters for CWS and MWS conditions. (PDF)

S1 Table Overview of the body points obtained with the multi-Kinect v2 set-up and the Optotrak system. (PDF)

S1 Video Body point’s time series obtained with the multi-Kinect v2 set-up and the Optotrak system of a single representative trial during the CWS condition of the 10MWT. (MP4)
