# Supplementary material for: Kinematic Validation of a Multi-Kinect v2 Instrumented 10-Meter Walkway for Quantitative Gait Assessments
Source: PLoS One. 2015 Oct 13;10(10):e0139913. doi: 10.1371/journal.pone.0139913 (PMC4603795; doi:10.1371/journal.pone.0139913)
Supplement: S1 Fig — (PDF) [file pone.0139913.s002.pdf]

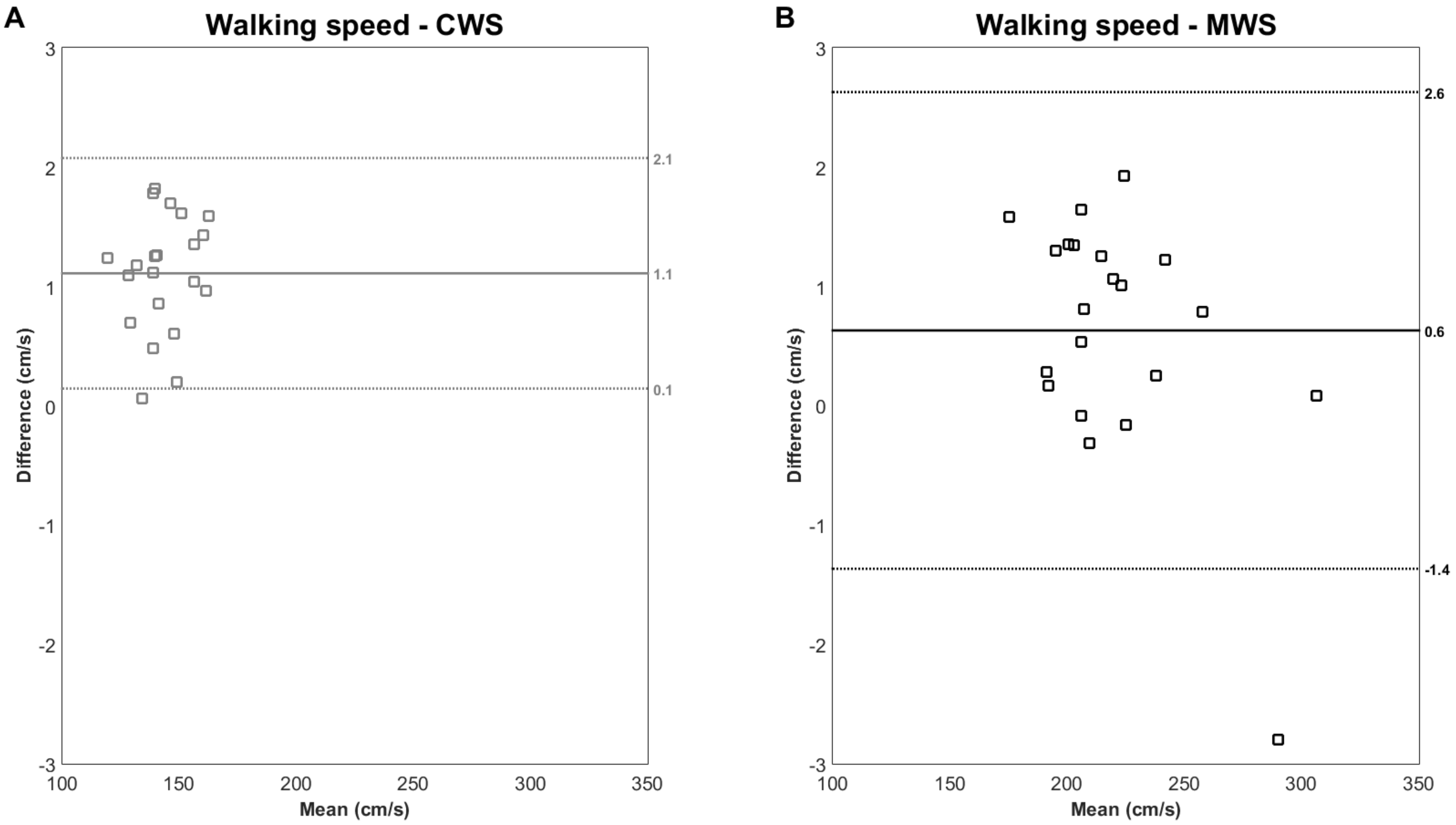


Figure 1: Bland-Altman plot for walking speed during the comfortable walking speed (CWS; A) and maximum walking speed (MWS; B) condition. Solid lines represent biases between the two motion registration systems. Dashed lines represent the 95% limits of agreement.

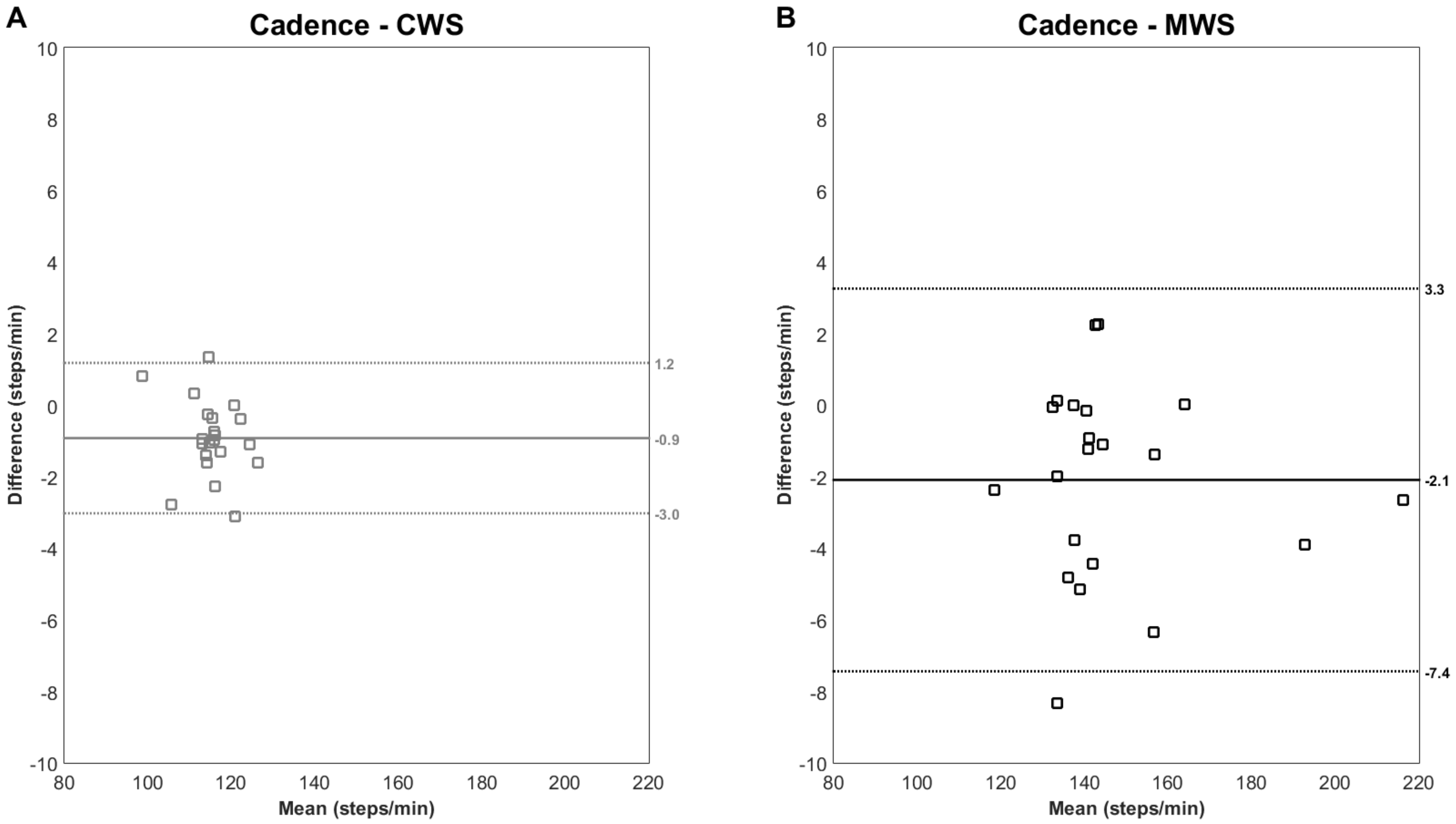


Figure 2: Bland-Altman plot for cadence during the comfortable walking speed (CWS; A) and maximum walking speed (MWS; B) condition. Solid lines represent biases between the two motion registration systems. Dashed lines represent the 95% limits of agreement.

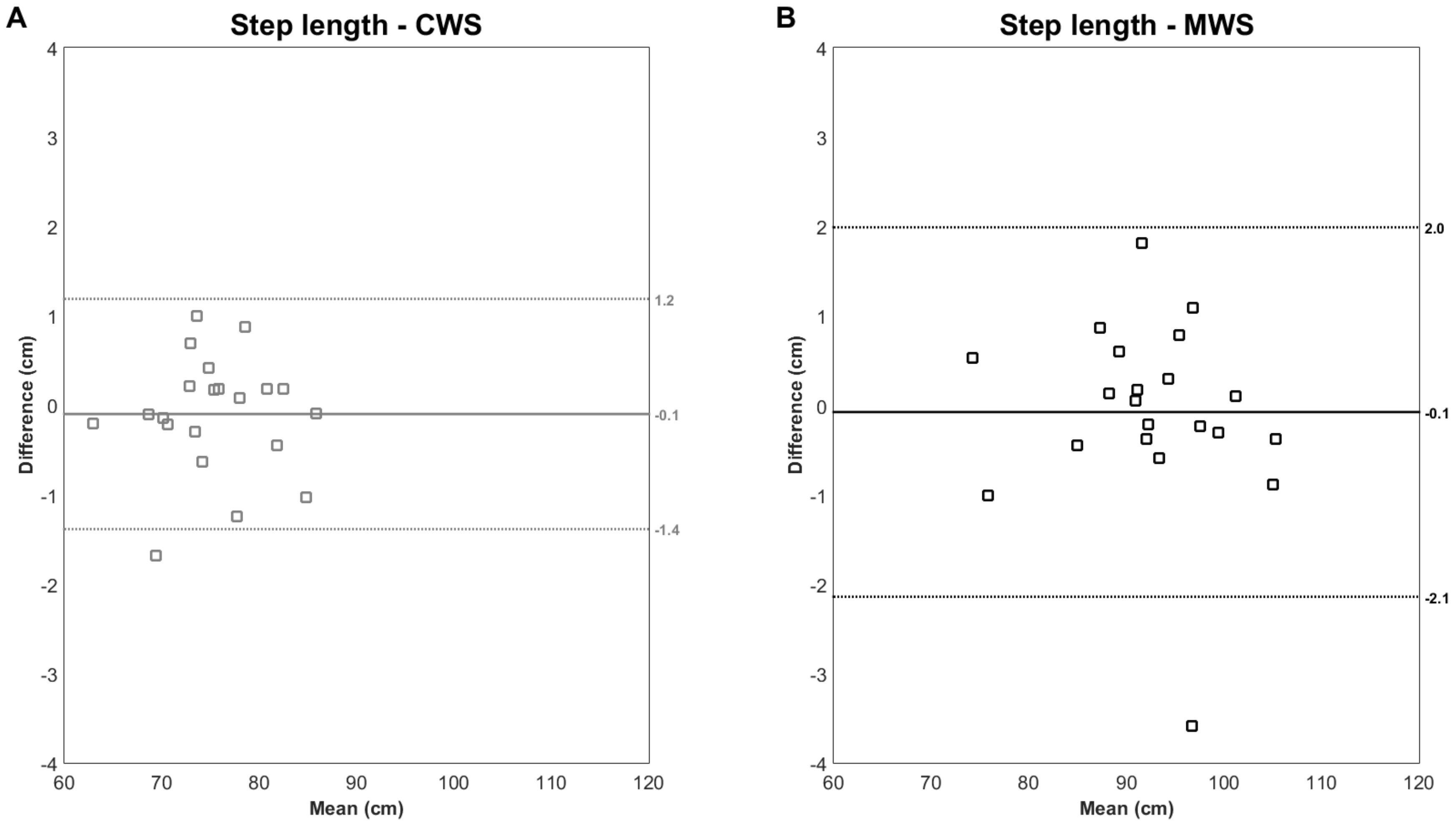


Figure 3: Bland-Altman plot for step length during the comfortable walking speed (CWS; A) and maximum walking speed (MWS; B) condition. Solid lines represent biases between the two motion registration systems. Dashed lines represent the 95% limits of agreement.

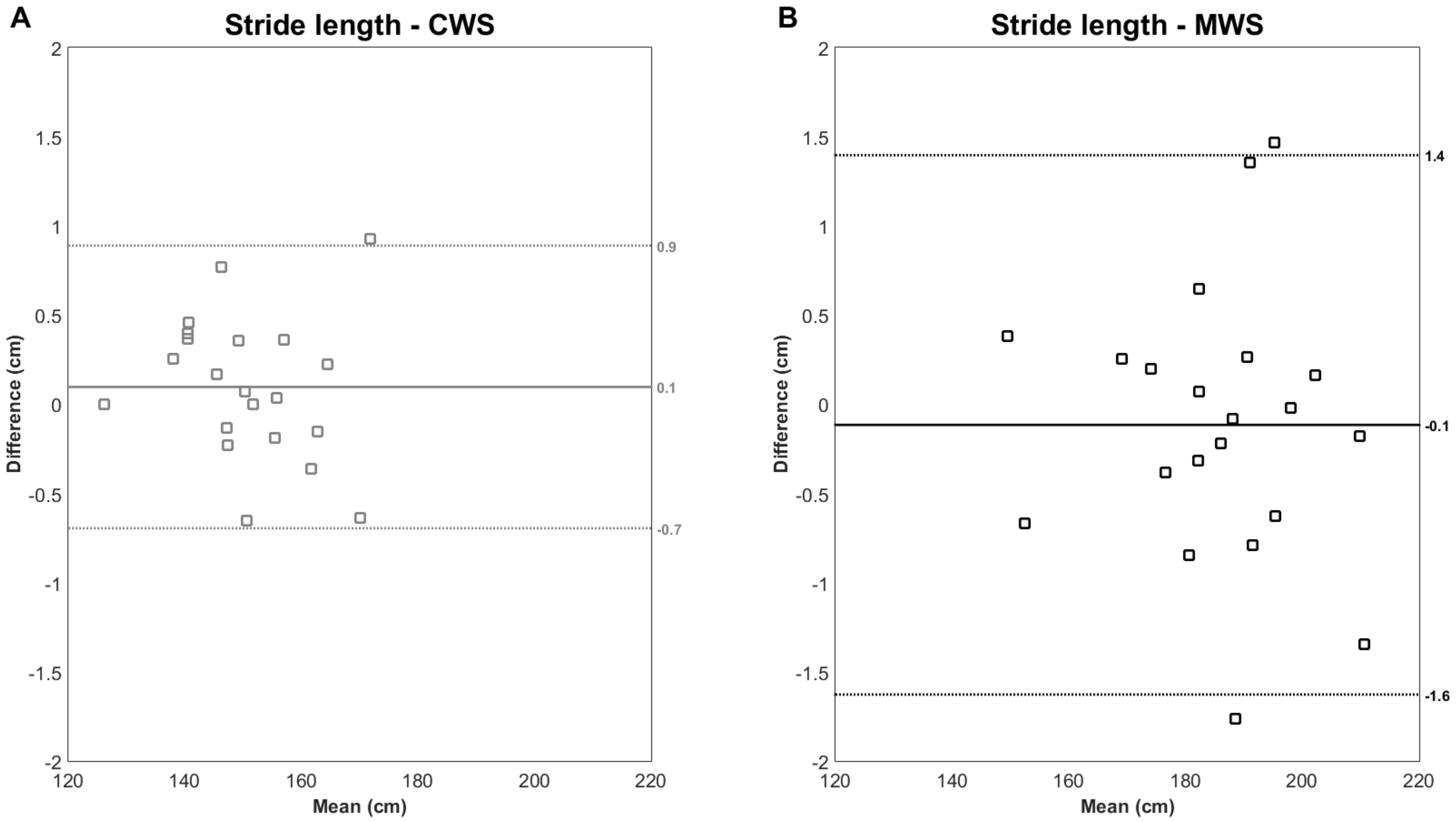


Figure 4: Bland-Altman plot for stride length during the comfortable walking speed (CWS; A) and maximum walking speed (MWS; B) condition. Solid lines represent biases between the two motion registration systems. Dashed lines represent the 95% limits of agreement.

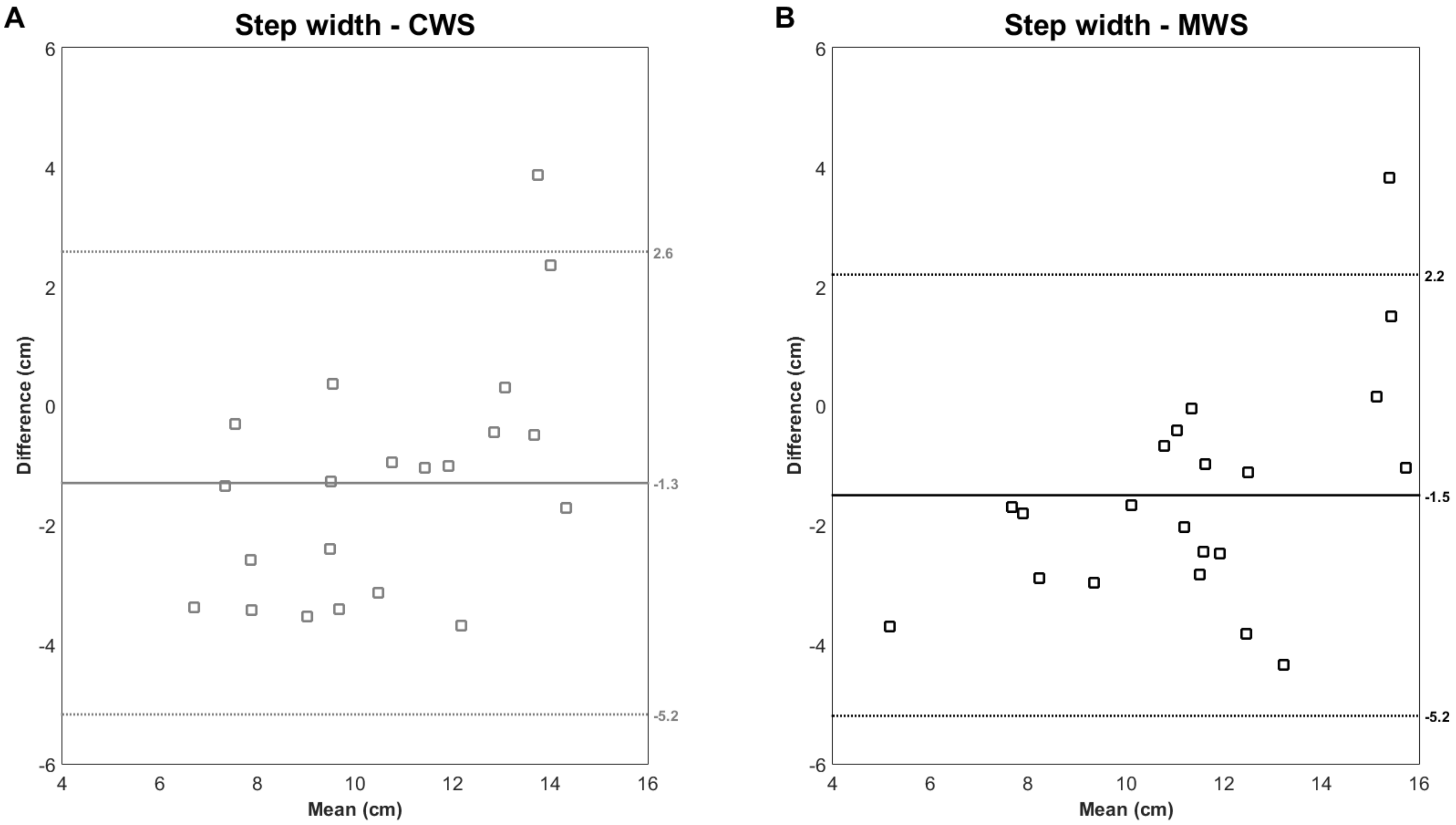


Figure 5: Bland-Altman plot for step width during the comfortable walking speed (CWS; A) and maximum walking speed (MWS; B) condition. Solid lines represent biases between the two motion registration systems. Dashed lines represent the 95% limits of agreement.

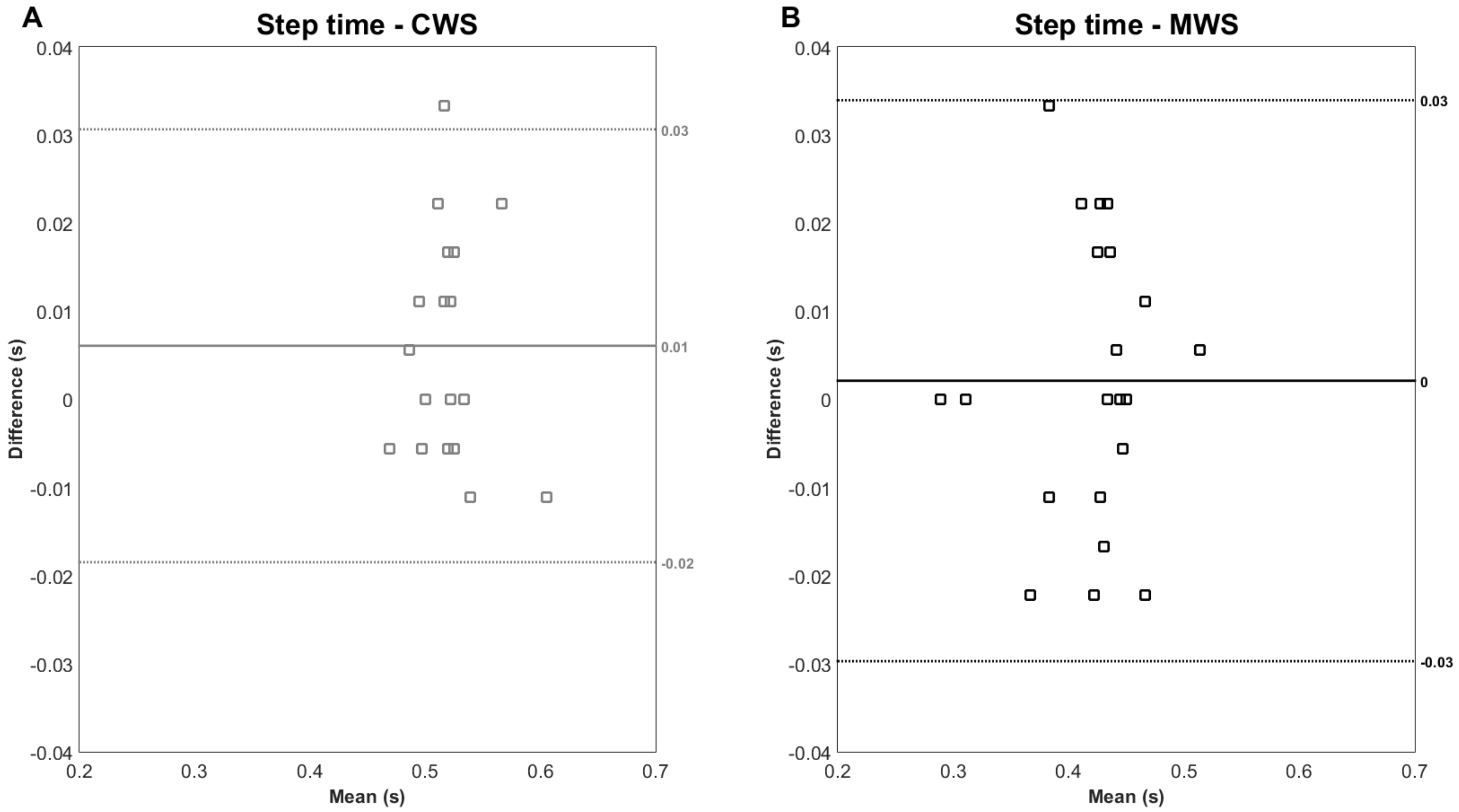


Figure 6: Bland-Altman plot for step time during the comfortable walking speed (CWS; A) and maximum walking speed (MWS; B) condition. Solid lines represent biases between the two motion registration systems. Dashed lines represent the 95% limits of agreement.

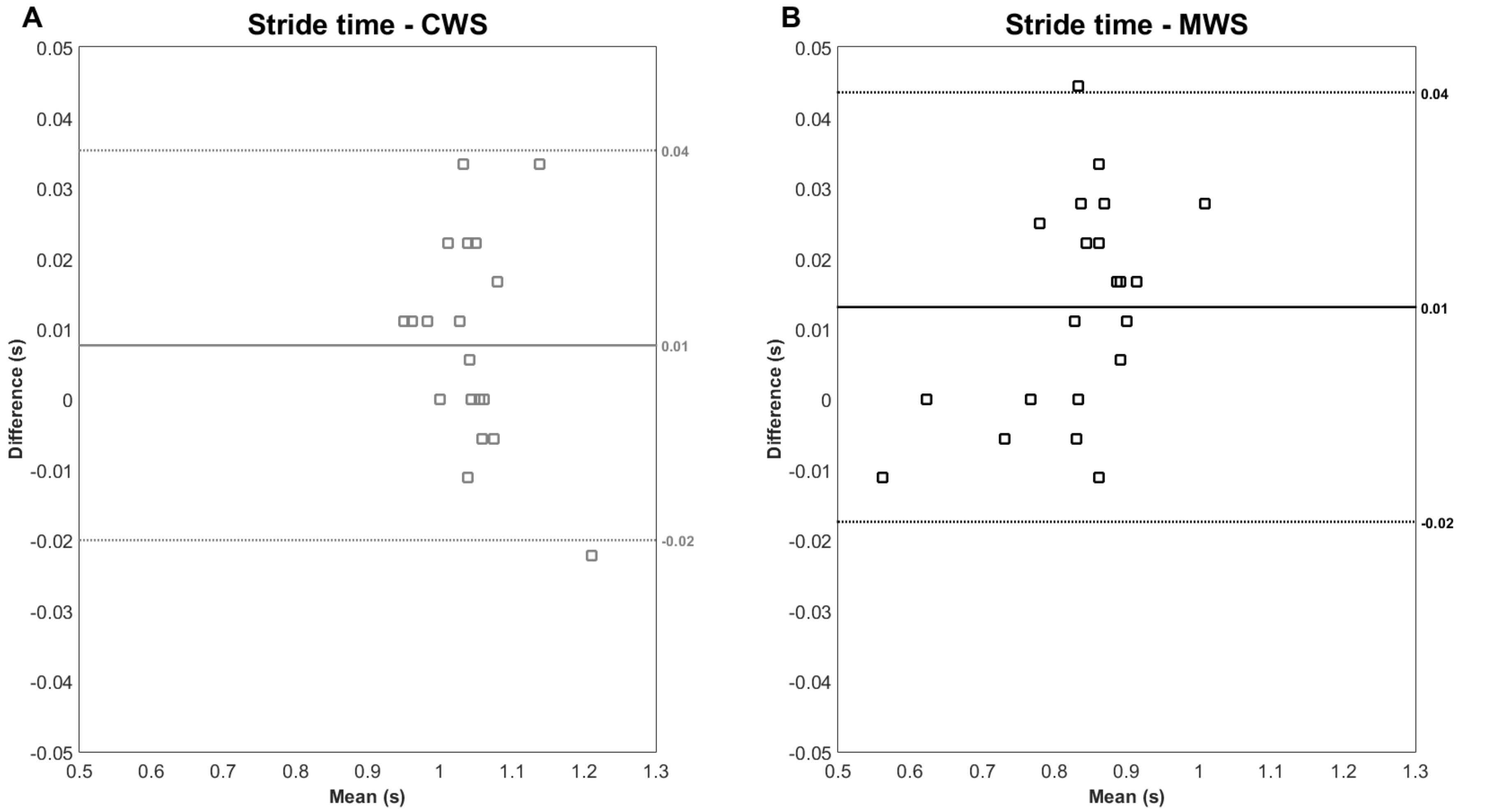


Figure 7: Bland-Altman plot for stride time during the comfortable walking speed (CWS; A) and maximum walking speed (MWS; B) condition. Solid lines represent biases between the two motion registration systems. Dashed lines represent the 95% limits of agreement.
