# Supplementary material for: Kinematic Validation of a Multi-Kinect v2 Instrumented 10-Meter Walkway for Quantitative Gait Assessments
Source: PLoS One. 2015 Oct 13;10(10):e0139913. doi: 10.1371/journal.pone.0139913 (PMC4603795; doi:10.1371/journal.pone.0139913)
Supplement: S1 Table — (PDF) [file pone.0139913.s003.pdf]

Overview of Kinect and Optotrak body points. For the latter, AP, ML and V position time series were computed from virtual markers and/or smart markers. In case of a single virtual marker or smart marker, the time series of that specific marker were taken as the time series of the associated body point. In case of multiple virtual markers and/or smart markers, the associated marker positions were averaged in all three directions for each time sample.

Kinect body point	Smart Marker Rigid Body position	Virtual marker position	Smart marker position
**Head**	Head	Nasion, inion and right and left ear	-
**Neck**	-	-	-
**Spine shoulder**	-	-	Sternum
**Spine mid**	-	-	-
**Spine base**	Lower abdomen	Right and left anterior superior and posterior superior iliac spine	-
**Shoulders**	Upper arms	Head of the humurus	-
**Elbows**	Upper arms	Medial and lateral epicondyles	-
**Wrists**	Forearms	Distal heads of the radius and ulna	-
**Hands**	-	-	Back of the hand
**Hand tips**	-	-	-
**Thumbs**	-	-	-
**Hips**	Upper legs	Trochantor major	-
**Knees**	Upper legs	Medial and lateral condyles	-
**Ankles**	Lower legs	Medial and lateral malleoli	-
**Feet**	Feet	Calcaneus	Head of the distal phalanx of the hallux
